# Three-Dimensional Bioprinting in Reconstructive Plastic Surgery: A Comprehensive Review

**DOI:** 10.3390/cells15171595

**Published:** 2026-09-02

**Authors:** Rahim Hirani, Sarina Iraj, Mathew Trandafirescu, Carter J. Boyd, Mill Etienne

**Affiliations:** 1 School of Medicine, New York Medical College, Valhalla, NY 10595, USA; 2 Hansjörg Wyss Department of Plastic Surgery, NYU Langone Health, New York, NY 10016, USA

**Keywords:** 3D bioprinting, reconstructive surgery, plastic surgery, tissue engineering

## Abstract

Three-dimensional (3D) bioprinting is an evolving biofabrication approach in regenerative medicine with the potential to overcome many limitations of conventional reconstructive techniques, including donor-site morbidity, limited tissue availability, and suboptimal restoration of form and function. Recent advances in biofabrication have accelerated the development of patient-specific living constructs for reconstructive applications. This narrative review synthesizes contemporary evidence on the use of 3D bioprinting in reconstructive surgery, emphasizing developments most relevant to plastic surgery. The current literature on bioprinting technologies, bioinks, tissue-specific applications, translational studies, and regulatory considerations was critically reviewed. Significant progress has been achieved in the bioprinting of skin, cartilage, bone, osteochondral tissues, vascularized constructs, and composite craniofacial tissues. Advances in extrusion-, inkjet-, laser-, and stereolithography-based printing, together with increasingly sophisticated natural and synthetic bioinks, have improved construct fidelity, cellular viability, and tissue-specific functionality. In situ bioprinting, patient-specific computer-aided design, and hybrid biomaterial strategies have further expanded the clinical potential of bioprinted tissues. Despite these advances, major barriers remain, including inadequate vascularization of large constructs, limited mechanical maturation of load-bearing tissues, manufacturing standardization, regulatory uncertainty, and the absence of robust long-term clinical outcomes. Three-dimensional bioprinting is enabling increasingly personalized tissue fabrication, although most applications remain preclinical. Clinical translation will require further advances in biomaterials, vascular engineering, manufacturing standardization, and regulatory science.

## 1. Introduction

Reconstructive surgery aims to restore the form and function of tissues damaged by trauma, congenital anomalies, tumor resection, or degenerative disease. For decades, autologous tissue grafts and vascularized flaps have served as the gold standard for soft- and hard-tissue reconstruction. However, these approaches carry well-recognized limitations, including donor site morbidity, limited tissue availability, prolonged operative times, and variable aesthetic and functional outcomes [1,2,3]. More broadly, the global demand for transplantable tissues and organs continues to far exceed the available supply. Although a record 173,727 solid organ transplants were performed worldwide in 2024, a profound shortage persists across all organ types, with kidney transplantation alone facing an estimated global deficit of more than 200,000 procedures [4,5]. These unmet clinical needs have accelerated efforts to develop engineered tissue substitutes capable of reproducing the structural, mechanical, and functional properties of native human tissues.

Three-dimensional (3D) bioprinting has emerged as a biofabrication technology at the intersection of regenerative medicine, materials science, and advanced manufacturing. By enabling the precise, layer-by-layer deposition of living cells, biomaterials, and bioactive molecules, 3D bioprinting facilitates the fabrication of patient-specific, multicellular tissues with complex architectures that recapitulate key structural and functional features of native tissue microenvironments [6,7]. Multiple bioprinting modalities, including extrusion-based, inkjet-based, laser-assisted, and stereolithographic techniques, offer distinct advantages in resolution, cell viability, and scalability. Collectively, these approaches have demonstrated the ability to generate tissue analogs of skin, cartilage, bone, vascular networks, and composite craniofacial tissues [8,9,10]. Concurrently, advances in bioink development have expanded the design space for tissue engineering. Bioinks incorporating natural polymers (e.g., collagen, alginate, gelatin) and synthetic polymers (e.g., polycaprolactone, polylactic acid) enable precise tuning of mechanical properties, biological activity, and degradation kinetics to meet the requirements of diverse regenerative applications [6].

These three fabrication strategies are frequently conflated under a single translational heading, yet they differ in regulatory classification, biological risk, and proximity to reconstructive practice. Acellular 3D-printed scaffolds contain no living cells at implantation and function as patient-specific osteoconductive or contour devices; examples include the 3D-printed calcium-phosphate implants described by Anderson et al. and the poly(glycerol sebacate) mandibular scaffolds reported by Yan et al. [11,12]. Cell-seeded scaffolds combine a prefabricated acellular matrix with subsequently applied cells and therefore occupy an intermediate regulatory category. True cell-laden bioprinting deposits living cells within the bioink during fabrication. The investigational AuriNovo auricular construct is a 3D-bioprinted collagen hydrogel that encapsulates autologous chondrocytes and is a first-in-human example of this class; it remains without marketing approval [13]. The 4D-printed, near-infrared-responsive bone scaffold reported by Choudhury et al. is a deployable, self-fitting acellular construct evaluated in rabbit cranial defects and should not be interpreted as cell-laden bioprinting [14]. Throughout this review, these categories are distinguished rather than treated as a single translational continuum.

Within reconstructive surgery, 3D bioprinting has shown promise across a broad spectrum of tissue applications. Skin bioprinting has evolved from simple epidermal sheets toward full-thickness, multilayered constructs incorporating dermal and vascular components, with in situ bioprinting strategies enabling the direct deposition of living tissues at wound sites [15,16]. Cartilage bioprinting has progressed through the development of organoid-based approaches and zonally organized constructs that aim to replicate the biomechanical and structural heterogeneity of native articular cartilage [17]. Bone regeneration strategies have leveraged composite scaffolds incorporating osteogenic and angiogenic cues to promote coordinated vascularized bone formation within critical-size defects [18,19]. Despite these advances, the creation of functional vascular networks remains one of the greatest challenges in tissue engineering, as inadequate microvascularization limits the survival, maturation, and integration of large bioprinted constructs [10,20]. In craniofacial reconstruction, bioprinting offers the potential for patient-specific solutions to complex zygomatic, orbital, nasal, and mandibular defects, with recent preclinical studies demonstrating the feasibility of intraoperative bioprinting of composite hard and soft tissues [16,21].

Despite these advances, the clinical translation of 3D bioprinted tissues remains in its infancy. Major barriers include the challenge of achieving adequate vascularization and innervation in large tissue constructs, the lack of standardized bioink formulations and manufacturing protocols, insufficient long-term data on in vivo tissue maturation and integration, and the absence of well-defined regulatory frameworks from agencies such as the U.S. Food and Drug Administration and the European Medicines Agency [6,20,22]. As the field transitions from proof-of-concept studies toward early clinical applications, rigorous preclinical validation, standardized manufacturing practices, and interdisciplinary collaboration will be essential to bridge the gap between laboratory innovation and routine clinical use. After outlining the review methodology, this review synthesizes the current state of 3D bioprinting in reconstructive surgery across skin, cartilage, bone and osteochondral tissue, facial and craniofacial reconstruction, vascularized constructs, and innervation, and it compares emerging bioassembly strategies. For each domain, the review synthesizes recent advances in bioprinting techniques, bioink design, and preclinical and emerging clinical evidence, while critically evaluating the scientific, technical, and translational challenges that remain. Representative studies across each tissue domain are summarized in Table 1, and a comparative, clinically oriented overview of principal bioink classes is provided in Table 2. Printing modalities and translational readiness by tissue application are compared in Table 3 and Table 4. By synthesizing the current evidence and highlighting remaining knowledge gaps, this review aims to identify the principal requirements for advancing 3D bioprinting from experimental investigation to clinically viable reconstructive therapies.

## 2. Methodology

This narrative review was informed by searches of PubMed/MEDLINE, Scopus, and Web of Science covering January 2012 through June 2026, with emphasis on 2018–2026. Search terms combined bioprinting and reconstructive vocabulary, including 3D bioprinting, bioink, extrusion, inkjet, laser-assisted, stereolithography, digital light processing, FRESH, aspiration-assisted, chaotic printing, coaxial, skin, cartilage, bone, osteochondral, craniofacial, vascularization, innervation, reconstructive surgery, and plastic surgery. Inclusion was limited to peer-reviewed English-language primary studies and reviews relevant to reconstructive plastic surgery. Non-peer-reviewed sources were excluded from the efficacy synthesis and used only when uniquely needed to document regulatory or first-in-human status, in which case they are identified as such. Table 1 presents representative peer-reviewed studies from 2021–2026 organized by tissue domain and is not an exhaustive catalog. Because this was a narrative rather than systematic review, study selection was intended to provide representative coverage rather than exhaustive evidence capture. Studies included in Table 1 were prioritized when they represented a distinct fabrication strategy, reported comparative or functional outcomes, used clinically relevant in vivo models, or reflected the highest level of translational evidence within a tissue domain; select acellular 3D-printed comparators were retained only when needed to clarify translational boundaries or current standards of care.

## 3. Bioinks and Biomaterials for Reconstructive Bioprinting

Bioink composition plays a critical role in determining construct printability, cellular fate, and the mechanical competence of bioprinted tissues, in concert with printing parameters, crosslinking strategies, construct architecture, and post-bioprint maturation. However, no single material simultaneously satisfies the competing demands of high cell viability, shape fidelity, and physiologically relevant stiffness [36,37]. Bioink selection is therefore inherently tissue specific. Rather than seeking a single optimal material, contemporary strategies increasingly combine a cell-instructive hydrogel with a load-bearing synthetic scaffold to exploit the complementary strengths of each [37,38]. Natural polymers provide biological cues but are mechanically weak, whereas synthetic thermoplastics provide structural integrity but lack intrinsic bioactivity; reconciling this trade-off remains the core materials challenge of the field.

Among natural hydrogels, collagen is the most physiologically representative dermal bioink, constituting the majority of the cutaneous extracellular matrix, but its slow gelation and low compressive modulus (typically 0.1–2 kPa) preclude standalone use in load-bearing constructs [37]. Alginate offers the opposite profile: rapid ionic crosslinking with calcium chloride confers excellent shape fidelity, but the absence of mammalian cell-adhesion motifs limits long-term integration unless the polymer is functionalized with arginine–glycine–aspartate peptides or blended with gelatin [39]. Gelatin methacryloyl (GelMA) has become one of the most widely used bioinks because its photocrosslinkable backbone permits tunable stiffness across roughly 0.5–100 kPa and retains the cell-adhesive sequences of native gelatin, although photoinitiator cytotoxicity at higher concentrations remains a concern [37]. Decellularized extracellular matrix (dECM) bioinks occupy a distinct niche because they retain tissue-specific growth factors and adhesion ligands that single-component hydrogels cannot reproduce. Early comparative work found that adipose, cartilage, and cardiac dECM bioinks sustained higher cell viability and more tissue-specific gene-expression patterns than collagen controls, supporting the broader principle that the tissue origin of the extracellular matrix, in addition to its biochemical composition, helps shape cellular phenotype [40]. Silk fibroin extends the attainable mechanical range, with compressive moduli reported between 10 and 500 kPa depending on β-sheet crystallinity, and fast-setting enzymatically crosslinked formulations have been printed into patient-specific, memory-shape implants [41]. Hyaluronic acid, a glycosaminoglycan abundant in cartilage and dermal matrix, contributes anti-inflammatory signaling and supports progenitor chondrogenesis; in its unmodified form it degrades rapidly and lacks mechanical strength, but methacrylation enables ultraviolet crosslinking with tunable moduli and improves the printability and filament stability of blended bioinks without impairing zone-specific differentiation [39,42]. Fibrin, formed by the rapid thrombin-mediated polymerization of fibrinogen, mimics the provisional wound matrix and gels within seconds at physiological conditions, making it the carrier of choice for in situ skin deposition and a useful vehicle for the sustained release of osteogenic factors in bone applications [43,44].

Despite their excellent biological properties, the principal limitation of hydrogel bioinks is inadequate mechanical strength, particularly for load-bearing applications. This challenge is most directly addressed through hybrid fabrication with polycaprolactone (PCL). As a melt-printable, semi-crystalline thermoplastic with a compressive modulus in the cortical-bone range and a degradation profile spanning two to four years, PCL provides the structural scaffolding into which softer cell-laden hydrogels are co-deposited [37]. PCL microfiber reinforcement has been shown to substantially increase the compressive modulus of alginate constructs, raising construct stiffness into the range of native articular cartilage [39]. This composite design philosophy underpins most clinically oriented constructs and reframes the bioink question from a search for a single ideal material toward the rational spatial assignment of distinct materials to distinct mechanical and biological roles. The same logic extends to fully resorbable synthetics such as the polylactide and polyglycolide copolymers, whose hydrolytic degradation can be tuned from weeks to months by monomer ratio and which are widely incorporated into composite bone scaffolds where complete resorption and replacement by host tissue is desirable [37]. A comparative, application-oriented summary of the principal bioink classes is provided in Table 2. Despite these advances, clinical translation remains constrained by the lot-to-lot variability of dECM preparations, the cytotoxicity ceiling of photoinitiators, manufacturing and regulatory challenges associated with living cell-based products, and the near-total absence of long-term in vivo performance data for cell-laden bioinks implanted into load-bearing human tissues [22].

## 4. Emerging and Non-Canonical Bioinks

Beyond collagen, gelatin methacryloyl (GelMA), alginate, decellularized extracellular matrix, and polycaprolactone, several non-canonical bioink classes have expanded the design space without yet entering reconstructive practice. Self-assembling peptide bioinks form nanofibrous scaffolds that support organotypic culture under physiologic conditions [45]. Supramolecular and dynamically crosslinked hydrogels introduce reversible bonds that permit self-healing after extrusion and may reduce nozzle-induced damage. Conductive and 4D-responsive hydrogels, including the multifunctional system reported by Joshi et al., couple printability with electrical or stimulus-responsive function relevant to innervated constructs [46]. Jammed microgel, or granular, inks flow under shear and rapidly recover a solid-like state after deposition, enabling high-resolution printing of soft, cell-protective matrices [47]. High-cell-density formulations, approaching 1.5 × 10^8^ cells/cm^3^ in granular spheroid systems, more closely approximate native tissue cellularity than conventional dilute hydrogels [33]. None of these materials has been used in an approved reconstructive implant.

## 5. Biological Consequences of Printing

Post-print viability is an incomplete success metric. Extrusion through a narrow nozzle imposes shear and extensional stresses that can compromise stem-cell integrity even when immediate viability remains high. Extrusion pressure and nozzle diameter and overall geometry further determine the magnitude and duration of shear exposure; higher pressure and narrower nozzles can improve resolution but increase the risk of cell injury and phenotypic alteration [48]. Photocrosslinking of GelMA and related methacrylated inks introduces photoinitiator and ultraviolet or visible-light exposure that may alter phenotype independently of acute cytotoxicity [37]. Thermal interfaces with melt-extruded polycaprolactone can locally injure adjacent cells. Longer-term consequences—stable lineage commitment, inflammatory activation, matrix quality, and in vivo function—are reported far less consistently than day-0 viability and should be required endpoints in reconstructive studies [22,48].

With respect to reconstructive plastic surgery, cell-laden collagen has reached investigational human use in auricular reconstruction, whereas acellular ceramic-composite and resorbable polyester implants used in craniofacial bone follow a separate 3D-printed device pathway [11,12,13]. GelMA, alginate, decellularized extracellular matrix, peptide, conductive, self-healing, and granular inks remain preclinical. High-cell-density and spheroid-based formulations remain experimental and have not been evaluated in large-animal reconstructive models at surgically relevant scale.

## 6. Skin Bioprinting

Skin is a major translational target in bioprinting because of its layered yet comparatively accessible architecture and the acute clinical need in burn and chronic-wound care. The field has advanced along two converging trajectories: the fabrication of increasingly faithful multilayered constructs and the development of in situ deposition directly within the wound bed. In situ bioprinting represents the more clinically proximate strategy; Albanna et al. integrated wound scanning with layer-by-layer deposition of autologous fibroblasts and keratinocytes in a fibrinogen–collagen carrier, accelerating re-epithelialization, reducing wound contraction, and promoting organized collagen deposition with mature vasculature at eight weeks in porcine full-thickness wounds [43]. Subsequent robotic systems have extended this concept. An adaptive, multi-degree-of-freedom in situ platform deposited stem-cell bioinks that regenerated not only epidermis and dermis but also functional appendages, including hair follicles and sebaceous glands, in a murine model [49]. Similarly, a robot-assisted platform using GelMA-encapsulated epidermal stem cells and skin-derived precursors achieved regeneration of hair-follicle-bearing skin [24]. The clinical appeal of in situ bioprinting lies in its ability to eliminate the ex vivo culture, transport, and grafting steps required for conventional tissue-engineered skin, while conforming precisely to irregular wound geometry and allowing tissue maturation within its native physiological environment.

A proposed advantage of bioprinted skin over split-thickness skin grafting is the potential to improve dermal architecture. In preclinical comparisons, split-thickness grafts showed parallel, scar-pattern collagen, whereas bioprinted constructs more closely recapitulated native basket-weave architecture; Jorgensen and colleagues quantified this directly in full-thickness wound models [50]. The same group advanced toward anatomical fidelity with a six-cell-type tri-layer construct incorporating epidermal, dermal, and hypodermal compartments together with melanocytes and endothelial cells, which on transplantation promoted rapid vascularization, rete ridge formation, and reduced fibrosis relative to graft controls in both murine and porcine models [23]. Vascularization strategies have been layered onto similar multi-bioink dermal constructs; GelMA/hyaluronic acid/fibrin composites seeded with human adipose-derived microvascular fragments and dermal fibroblasts have been reported to accelerate wound closure and vascularization relative to controls [25], and acellular dermal matrix combined with GelMA and a vascular endothelial cell layer has similarly supported re-epithelialization and angiogenesis in full-thickness murine wounds [26]. The addition of pigmentary, neural, and adnexal components marks an important step beyond earlier epidermal–dermal bilayers and simpler functional skin equivalents previously validated in vivo [51]. Laser-assisted printing has provided an alternative, nozzle-free deposition route capable of high-resolution cellular patterning of skin, while amniotic-fluid-derived stem cells have been shown to accelerate large-wound healing through paracrine rather than structural mechanisms [52,53]. Collectively, these studies illustrate the progressive evolution of bioprinted skin, from acellular dressings and epidermal sheets to vascularized, pigmented, innervated multilayer constructs capable of supporting skin appendages. Despite this increasing biological sophistication, the critical challenge remains demonstrating durable function and integration under the clinically relevant conditions of large, contaminated burn wounds rather than the controlled geometry of experimental excisional wound models.

The defining limitation of bioprinted skin remains vascularization, as constructs thicker than approximately 2 mm fail without perfusion. One strategy has been the incorporation of endothelial cells and pericytes into collagen-based dermal constructs, enabling the self-assembly of functional microvascular networks. These networks inosculated with host vessels and became perfused within four weeks, while pericyte inclusion further enhanced vascularization and epidermal maturation [54]. This self-assembly approach circumvents the resolution barrier of directly printing capillary-caliber vessels. The incorporation of skin appendages has likewise progressed, with dermal papilla and endothelial spheroids guiding follicular downgrowth in printed constructs [55]. Nonetheless, substantial translational barriers remain. To our knowledge, no bioprinted skin construct has been evaluated in a completed randomized human trial, and scale-up to extensive burns, xenogeneic matrix components, sterility assurance, and the limited shelf life of cell-laden bioinks continue to impede routine clinical implementation [56,57].

Current evidence highlights distinct limitations of the two dominant skin-bioprinting strategies. Intraoperative or in situ deposition places cells directly onto the wound and is attractive for irregular defects, yet the supporting evidence is confined to murine and porcine models and has not been tested in large, contaminated human burns [3,56,58]. Prefabricated, multilayer constructs can incorporate appendages and preformed microvasculature, but they have not been produced at the surface area required for major burn coverage and have not been compared head-to-head with split-thickness skin grafting in published human studies [3,56]. Until such trials exist, bioprinted skin should be viewed as an investigational adjunct rather than a replacement for autograft or flap reconstruction.

## 7. Cartilage Bioprinting

Cartilage is an attractive bioprinting target because it is avascular and alymphatic, eliminating the perfusion requirement that constrains most thick tissues. However, its complex zonal organization and demanding biomechanical properties make faithful reconstruction deceptively difficult. The choice of bioink strongly influences the resulting cartilage phenotype: in a systematic comparison, Daly et al. found that alginate and agarose supported hyaline-like, type II collagen–dominant cartilage, whereas GelMA and polyethylene-glycol-based inks favored a fibrocartilaginous, mixed type I and type II phenotype, despite comparable post-printing cell viability across materials [39]. Cell source is equally important. Articular cartilage–derived progenitor cells have outperformed mature chondrocytes in neocartilage production, with lower expression of the hypertrophy marker type X collagen and higher lubricin production, suggesting a stable superficial-zone phenotype suitable for the articulating surface [42]. Induced pluripotent stem cells offer a theoretically unlimited autologous source. When co-printed with irradiated chondrocytes in a nanocellulose–alginate bioink, iPSCs have demonstrated chondrogenic differentiation and hyaline cartilage matrix production, although concerns regarding tumorigenicity and the safe control of pluripotent cell differentiation remain important barriers to clinical translation [59].

Recapitulating the native zonal architecture of articular cartilage, rather than producing homogeneous tissue, has emerged as the principal structural objective of cartilage bioprinting. Daly and Kelly directed the self-organization of chondrocyte and mesenchymal stem cell spheroids within printed polymeric microchambers to generate depth-dependent collagen fiber orientation that closely mirrored native cartilage, a hierarchical structural feature not achieved with conventional cast hydrogels [60].

Sun and colleagues coupled gradient architecture with spatially controlled growth-factor delivery, releasing insulin-like growth factor 1 superficially and transforming growth factor β in the deep zone, and demonstrating regeneration of anisotropic, zonally lubricated cartilage in rabbit knee defects over six months that significantly outperformed single-factor controls [61]. More recently, a digital-light-processing approach used three photocrosslinkable, cell-laden bioinks (methacrylated collagen, hyaluronic acid, and mineralized collagen) to fabricate a covalently bonded, tri-layer osteochondral construct that achieved near-complete cartilage and subchondral bone restoration in a rat osteochondral defect model at 12 weeks [27], extending the gradient-architecture concept from cartilage alone to the full osteochondral unit. For facial cartilage, tissue-specific dECM has shown advantages over more generic hydrogel formulations. A photocrosslinkable auricular cartilage dECM bioink supported type II collagen and glycosaminoglycan production in anatomically accurate ear constructs and improved phenotype retention compared with gelatin-based controls [62]. Articular and facial cartilage impose different design requirements: articular reconstruction is dominated by the demand for zonal biomechanical function and load bearing, whereas auricular and nasal reconstruction prioritize the faithful reproduction of intricate three-dimensional contours, and the optimal bioink and fabrication strategy diverge accordingly. Heterocellular bioink design combines chondrocytes with supporting mesenchymal or progenitor populations to stabilize the chondrogenic phenotype, suppress hypertrophic differentiation, and accelerate matrix deposition. Early composite scaffolds incorporating PCL-reinforced, cell-laden alginate established the feasibility of this mechanically reinforced, heterocellular approach for cartilage tissue engineering [63,64]. These findings indicate that successful cartilage bioprinting depends on integrating spatial architecture, biomaterial composition, and cellular organization to reproduce the structural and functional heterogeneity of native cartilage.

The principal barrier to the clinical translation of cartilage bioprinting remains mechanical maturation. Bioprinted constructs typically achieve compressive moduli of 10–200 kPa in vitro, an order of magnitude below the 0.5–2 MPa of native articular cartilage, and PCL reinforcement represents an important strategy to partially bridge this deficit [39]. Although dynamic bioreactor conditioning accelerates matrix-driven stiffening, few studies incorporate this step before implantation, and the mismatch between the prolonged maturation required for functional tissue development and the immediate load-bearing demands of clinical implantation remains unresolved. Human evidence remains limited but is no longer absent. A 2023 single-arm, open-label study reported 10 patients with knee osteoarthritis treated with 3D-bioprinted micronized adipose tissue and allogeneic cartilage-matrix grafts on a PCL mold, with clinical, MRI, arthroscopic, and histologic follow-up to 12 months; the small uncontrolled design precludes comparison with established cartilage-restoration procedures [65]. Auricular and nasal reconstruction remain mechanically more tractable because contour fidelity, rather than immediate load bearing, is the primary requirement.

These mechanical and phenotypic constraints explain why cartilage strategies that succeed in vitro often fail under clinically relevant conditions. Hydrogel-only constructs preserve chondrocyte viability but cannot match the modulus of native articular cartilage, whereas polycaprolactone-reinforced hybrids improve stiffness at the expense of a non-native, slowly degrading polymer phase [39]. Phenotype is equally decisive: alginate and agarose favor hyaline-like matrix, whereas GelMA and polyethylene-glycol inks drift toward fibrocartilage despite comparable viability [39]. Induced pluripotent stem cells expand the cell-source pool but introduce tumorigenicity and differentiation-control risks that remain unresolved for implantation [59]. Facial cartilage remains mechanically more tractable than articular cartilage because auricular and nasal reconstruction demand contour more than immediate load bearing, yet even there the comparator remains autologous rib or native cartilage graft, against which no bioprinted implant has published long-term comparative outcomes [13].

Future progress will depend not only on improving bioinks but also on accelerating construct maturation so that engineered cartilage can meet the mechanical demands of implantation at the time of surgery.

## 8. Bone and Osteochondral Bioprinting

Bone bioprinting must reconcile two competing requirements: immediate mechanical stability sufficient for load bearing and a vascularized, osteoconductive environment that supports remodeling. An important advance came with the development of the Integrated Tissue–Organ Printer (ITOP), which simultaneously deposited a PCL framework, sacrificial Pluronic microchannels, and a cell-laden hydrogel to produce patient-scale mandibular and calvarial constructs. Following implantation, these constructs formed vascularized bone in vivo over five months without developing a necrotic core, with the printed microchannel network maintaining cell viability by facilitating nutrient diffusion [66]. Building on this platform, Lee and colleagues integrated computed-tomography-based patient-specific design with a biomimetic dense-outer, porous-inner scaffold architecture to enhance craniofacial bone regeneration in an orthotopic model compared with homogeneous controls [67].

These studies show the evolution of bone bioprinting from proof-of-concept construct fabrication toward biomimetic scaffold design that increasingly reproduces both the structural hierarchy and biological function of native bone.

Because mineralized scaffolds alone cannot regenerate critical-size defects, controlled growth-factor delivery has become an integral component of bone bioprinting. Sustained bone morphogenetic protein 2 (BMP-2) delivery has been achieved by encapsulating the growth factor in gelatin microparticles within alginate bioinks, enabling release over three weeks with spatial control and resulting in significantly greater ectopic bone formation than unloaded scaffolds [44]. More recent thermoresponsive strategies have extended this principle: a poly(organophosphazene)-based bioink tunable in stiffness across 5–37 °C enabled sustained co-release of BMP-2 and TGF-β1 and significantly improved bone regeneration in a rat calvarial defect [30], while hybrid bioinks combining thermosensitive PLGA/hydroxyapatite/polyethylene-glycol microparticles with a cell-laden ECM-based hydrogel produced mechanically robust, vascularized bone with superior regeneration compared with acellular controls in a rat femoral defect [29]. Successful regeneration of critical-size bone defects requires the coordinated development of both bone and its supporting vasculature. Consequently, dual-factor delivery strategies have become central to contemporary bone bioprinting. Spatiotemporally controlled co-delivery of BMP-2 and vascular endothelial growth factor has repaired large weight-bearing defects more effectively than single-factor delivery, reinforcing the importance of vascularization as a rate-limiting step in large-defect bone regeneration [68]. Naturally derived additives have also been explored as osteogenic bioink components; a GelMA bioink blended with amphibian skin-secretion mucin achieved a higher new bone volume fraction than a GelMA-only control in a rat cranial defect [31]. The inorganic phase remains important for reproducing native bone mechanics and mineral composition. Calcium phosphate ceramics, including hydroxyapatite and β-tricalcium phosphate, mimic native bone mineral and improve osteoinductivity. Clinical-scale, personalized calcium phosphate scaffolds have been printed and validated for alveolar reconstruction in preclinical models [11]. Similarly, nano-hydroxyapatite incorporation into gellan gum/polyvinyl alcohol bioinks has been shown to improve printing accuracy and reduce degradation rate in vitro [32].

The dominant unsolved problem in bone bioprinting is the vascularization of constructs beyond the diffusion limit, as cores thicker than approximately 2 mm undergo avascular necrosis without pre-vascularization. Sequential rather than simultaneous delivery of angiogenic and osteogenic factors, and attention to their relative ratios, has improved outcomes in several systems [68]. The relative ratio of the two factors is itself consequential, with osteogenically favorable ratios promoting bone formation and disproportionately high angiogenic loading capable of inhibiting bone formation, underscoring that growth-factor bioprinting is a problem of quantitative spatial control rather than mere co-delivery [68]. Granular, prevascularized bioink strategies represent a complementary approach: prevascularized mesenchymal spheroids embedded at high density in an extracellular-matrix-based granular bioink have been shown to self-organize into interconnected vascular networks through angiogenic sprouting while supporting osteogenic differentiation [33]. Despite encouraging preclinical results, controlled human evidence for cell-laden bone bioprinting remains lacking, and regulatory barriers, including good manufacturing practice (GMP)-compliant cell expansion, xenogeneic bioink components, and the absence of standardized lot-release criteria for cell-laden constructs, continue to impede translation [69]. Standard of care remains vascularized bone flaps and, where appropriate, patient-specific acellular implants; murine and rat osteogenesis should not be equated with translational readiness in the absence of large-animal mechanical function, surgical handling, clinically relevant controls, and adequate follow-up [69].

### Facial and Craniofacial Reconstruction

Facial and craniofacial defects, whether congenital, oncologic, or traumatic in origin, present a distinct reconstructive challenge because they require restoration of complex hard-tissue contours, soft-tissue coverage, and aesthetic symmetry, often within a single operative field. Unlike isolated skin, cartilage, or bone defects, craniofacial reconstruction frequently requires composite constructs that integrate osteogenic, chondrogenic, and dermal components across curved, patient-specific geometries such as the zygoma, orbital floor, nasal dorsum, and mandible. In this setting, 3D bioprinting offers a potential advantage because patient imaging can be converted into computer-aided designs that guide the fabrication of anatomically matched scaffolds and cell-laden constructs.

Intraoperative bioprinting is a potential strategy for craniofacial reconstruction because it allows the surgeon to deposit bioinks directly into an irregular defect rather than relying entirely on prefabricated constructs. This approach may be especially useful after trauma, tumor resection, or debridement, where the final defect geometry may differ from preoperative imaging. Moncal and colleagues demonstrated proof of concept for this strategy by intraoperatively bioprinting composite hard- and soft-tissue constructs directly onto combined calvarial and full-thickness skin defects in a rat model. The constructs incorporated an osteogenic hard-tissue ink and a separate skin-cell-laden soft-tissue bioink, achieving substantial bone coverage and wound closure within weeks [16]. This composite approach addresses a major limitation of isolated skin, cartilage, and bone engineering: craniofacial defects rarely respect tissue-plane boundaries, and clinically useful constructs will likely need to function as integrated multi-tissue units rather than single-material scaffolds.

Ex vivo, image-guided craniofacial constructs have also advanced toward clinically relevant workflows. Computed-tomography-derived biomimetic scaffolds with a dense outer shell and porous internal architecture have been shown to balance mechanical protection with vascular ingrowth and improve craniofacial bone formation compared with homogeneous scaffolds in an orthotopic model [67]. Personalized calcium phosphate scaffolds have likewise been printed at clinical scale for alveolar ridge reconstruction, highlighting the potential for imaging-based fabrication in craniofacial and dental rehabilitation [11]. In an 8-mm rabbit mandibular defect model, a 3D-printed porous poly(glycerol sebacate) scaffold promoted macroscopic bone healing, with greater new-bone width and thickness than empty controls at six weeks and no obvious infection or purulence [12]. These approaches are most relevant for mandibular, calvarial, orbital, zygomatic, and alveolar defects, where precise anatomic fit is essential for both function and appearance.

Cartilaginous facial structures represent another craniofacial application under investigation. Auricular and nasal reconstruction are driven primarily by contour fidelity, elasticity, and long-term shape retention rather than immediate load bearing. This makes them mechanically more tractable targets than articular cartilage, which must withstand substantial compressive forces. Tissue-specific bioinks, including cartilage-derived decellularized extracellular matrix and chondrocyte-laden hydrogels, may help preserve chondrogenic phenotype while allowing fabrication of patient-specific auricular or nasal geometries. The AuriNovo auricular reconstruction program illustrates this translational direction; its Phase 1/2a study was terminated by company decision, not for safety reasons, after enrollment of two participants, with no results posted, and AuriNovo has not received marketing approval [13]. Long-term durability, growth behavior, and comparative outcomes against conventional rib-cartilage reconstruction remain to be established.

For facial plastic and reconstructive surgery, the broader value of bioprinting lies in the convergence of imaging, computer-aided design, bioink engineering, and surgical implantation into a workflow that parallels existing virtual surgical planning. However, major barriers remain. Craniofacial constructs must integrate across curved, mechanically complex surfaces; maintain contour under functional loading; support rapid vascularization in potentially irradiated or contaminated wound beds; and, for soft-tissue reconstruction, ultimately restore sensation and possibly appendage-like structures. These challenges place craniofacial bioprinting at the intersection of cartilage, bone, skin, and vascularized composite tissue engineering. As a result, facial reconstruction may serve as an important proving ground for 3D bioprinting, but its routine clinical use will depend on solving the same problems that constrain the field more broadly: vascular integration, mechanical maturation, reproducible manufacturing, and long-term in vivo stability.

In current practice, complex facial defects are reconstructed with free tissue transfer, patient-specific acellular implants such as titanium or polyetheretherketone (PEEK), and autologous rib cartilage rather than living bioprinted constructs [1,21]. The 3D-printed poly(glycerol sebacate) mandibular scaffolds of Yan et al. and the calcium-phosphate implants of Anderson et al. are acellular, patient-specific devices and should not be counted as cell-laden bioprinting [11,12]. Until bioprinted composites demonstrate surgical handling, vascular integration, and contour stability comparable to these standards, they remain adjunctive research tools.

## 9. Vascularization Strategies

Vascularization is the major unresolved challenge of bioprinting clinically relevant tissues. Two length scales are often conflated. The oxygen-diffusion and capillary-sprout reach limit is on the order of 200 µm from the nearest perfused vessel; beyond this distance, cells become hypoxic. A second, geometric limit of approximately 2 mm describes the necrotic core that develops in unperfused hydrogel constructs. Printed channels, endothelialized channels, self-assembled microvasculature, host inosculation, and surgically anastomosable vessels are distinct biological endpoints and should not be treated as interchangeable [70]. Surgical anastomosis has been demonstrated in selected vascular-graft models, but these examples should be distinguished from vascularizing thick reconstructive tissues. Szklanny et al. directly anastomosed a bioprinted vascularized tissue-flap system to the rat femoral artery [71]; Dell et al. implanted a fully bioprinted aortic conduit by end-to-end anastomosis in rats [72]; and Zuo et al. reported large-animal anastomosis of a hybrid ePTFE graft in which adipose-derived stromal-cell bioink was printed onto the prosthetic lumen [73]. The latter is a bioprint-assisted hybrid vascular graft rather than a fully bioprinted living vessel. Thus, the unresolved problem is not surgical anastomosis per se, but scalable integration of a surgically accessible inflow/outflow conduit with dense, durable microvascular networks throughout thick reconstructive tissue. The foundational strategy is the use of sacrificial, or fugitive, inks to template perfusable channels. Carbohydrate-glass lattices have been used to generate endothelializable channels after dissolution, allowing primary hepatocytes in the construct core to retain metabolic function and directly linking perfusion to viability independent of matrix chemistry [36]. This approach was extended using bioprinted agarose template fibers within photocrosslinkable hydrogels to improve nutrient transport, while thermoreversible Pluronic F-127 emerged as a versatile fugitive ink [74]. In thicker constructs, co-printing a Pluronic vascular template within a cell-laden matrix generated vascularized tissues exceeding one centimeter that remained viable under perfusion for more than six weeks; by contrast, unperfused controls of equivalent thickness exhibited greater than 70% core cell death within twelve hours [75].

Recent advances have narrowed the gap between printed channels and functional microvasculature. The Sacrificial Writing Into Functional Tissue technique embedded vascular channels within a matrix of compacted organ building blocks approaching native cellular density, with perfused cardiac constructs demonstrating a more than twenty-fold increase in contractile force, thereby addressing the cell-density deficit of earlier sparse-hydrogel constructs [76]. The freeform reversible embedding of suspended hydrogels enabled collagen printing at approximately 20-micrometer resolution within a removable support bath, allowing fabrication of soft extracellular-matrix structures from capillary to organ scale [77,78]. Stereolithographic printing with biocompatible photoabsorbers, reported by Grigoryan and colleagues, produced entangled, topologically independent vascular networks difficult to achieve by extrusion, including a functional alveolar model capable of oxygenating red blood cells [79]. Coaxial and multi-nozzle approaches permit single-step fabrication of hollow, endothelialized macro-vessels through a core-sheath nozzle, although current resolutions confine these conduits to the macro-scale and capillary-caliber networks must still arise by secondary sprouting [80]. Extrusion of collagen/xanthan gum bioinks through a multi-head printer has similarly been used to spatially sandwich endothelial cells between fibroblast layers, generating interconnected capillary-like networks after culture [35]. Blend bioinks combining gelatin methacryloyl with vascular-supportive components have been used to print perfusable vascular conduits directly [81]. Personalized, perfusable thick cardiac constructs printed from patient-derived materials further illustrate the convergence of these strategies, demonstrating that anatomically specific, vascularized soft-tissue architectures can be fabricated from autologous matrix [82]. Beyond in vitro self-assembly, intraoperative approaches have coupled bioprinting with surgical micropuncture of host macrovasculature: in a rat hindlimb model, endothelial-cell-laden bioink combined with micropuncture produced significant increases in vessel density, vessel length, and endothelial marker expression relative to bioink alone; perfusion within the constructs confirmed functional microvascular anastomoses to the host [34]. These modalities are complementary rather than competing: extrusion offers scalability and multimaterial deposition, stereolithography offers topological complexity and speed, and embedded printing improves resolution in soft matrices. The optimal reconstructive strategy may integrate several modalities within a single construct.

Despite this progress, a decisive translational barrier persists. As articulated in a structured framework by Seymour et al., printed channels, endothelialized channels, self-assembled microvasculature, host inosculation, and surgically anastomosable vessels are distinct biological endpoints [70]. Angiogenic sprouting from printed channels remains limited to a reach of roughly 200 µm. Although surgical anastomosis has been demonstrated in selected small-animal bioprinted conduits and large-animal bioprint-assisted hybrid grafts [71,72,73], durable coupling of surgically accessible inflow/outflow vessels to capillary-scale networks throughout thick cell-laden reconstructive tissue remains unresolved in a large-animal or human model. The transition from printed vascular structure to integrated, tissue-wide perfusion therefore remains a major obstacle to fabricating thick, implantable reconstructive tissues.

## 10. Innervation

Sensory recovery is a defining outcome of successful skin and craniofacial reconstruction, yet neurite ingrowth, reinnervation density, and sensory testing are almost never reported as endpoints in bioprinting studies. Muller et al. generated an innervated tissue-engineered skin containing human sensory neurons and Schwann cells differentiated from induced pluripotent stem cells; neurite colonization of the construct required Schwann cells, underscoring that neuronal inclusion alone is insufficient [83]. Complementary work on 4D-printed multifunctional hydrogels has produced flexible nerve conduits capable of supporting peripheral repair, although these devices remain preclinical and have not been evaluated in a reconstructive soft-tissue model [46]. Until innervation is measured with the same rigor as vascularization, claims of functional skin or facial reconstruction will remain incomplete.

## 11. Emerging Bioprinting and Bioassembly Strategies

Several bioassembly strategies address limitations of conventional extrusion, inkjet, and light-based printing. Embedded printing, including freeform reversible embedding of suspended hydrogels (FRESH), supports printing of soft collagen constructs that would otherwise collapse, including components of the human heart, at the cost of a support bath that must be removed and of limited throughput [77,78]. Aspiration-assisted bioprinting positions prefabricated spheroids with high spatial precision; a freeform variant deposits those spheroids into a yield-stress gel, and the same platform has been used to assemble osteochondral interfaces and osteogenic spheroid constructs [84,85,86,87]. Scaffold-free tissue strands have been hybrid-printed into zonally stratified human articular cartilage, offering high cell density and native-like organization but requiring lengthy spheroid or strand maturation before implantation [88]. Continuous chaotic printing uses chaotic advection to generate ordered multilayer microstructures and multi-channel hydrogel filaments in a single step, enabling prevascularized muscle-like tissues without sequential coaxial alignment [89,90]. Coaxial and microfluidic printheads similarly create core–shell and perfusable filaments during extrusion [80,81].

Aspiration-assisted and spheroid-based assembly achieve high local cell density and tissue-like organization but scale poorly and consume substantial culture time. Chaotic and coaxial printing improve internal architecture and throughput but remain hydrogel-bound and have not produced surgically anastomosable vessels. FRESH improves geometric fidelity of soft matrices but does not itself solve vascular anastomosis or mechanical competence. No emerging modality has yet combined patient-scale geometry, surgical handling, large-animal mechanical function, and a clinically relevant control at adequate follow-up. Table 3 compares established and emerging modalities across resolution, viability, scalability, speed, cost, and the principal reconstructive limitation of each.

## 12. Clinical Trials and Translational Outcomes

The field has progressed from exclusively preclinical work toward limited human implantation, although the evidence remains sparse and heterogeneous. Human implantation, formal clinical investigation, regulatory designation, completed trials, and marketing approval are sequential and non-equivalent milestones. AuriNovo represents a first-in-human example: the Phase 1/2a study used a patient-specific 3D-bioprinted collagen hydrogel encapsulating autologous auricular chondrocytes for microtia reconstruction [13]. The sponsor publicly reported the first implantation in 2022 and FDA Orphan Drug and Rare Pediatric Disease designations [91]. ClinicalTrials.gov now lists the study as terminated by company decision, not for safety reasons, after enrollment of two participants, with no results posted; AuriNovo has not received FDA marketing approval [13]. Long-term shape stability and comparative outcomes against conventional rib-cartilage reconstruction have not been published in the peer-reviewed literature. For skin, intraoperative robotic bioprinting has been demonstrated by Albouy et al., who deposited cell-laden hydrogel directly onto third-degree burn wounds in a swine model under surgical conditions with enhanced re-epidermalization, establishing the feasibility of surgery-ready deposition outside a controlled laboratory environment [58].

Translational readiness differs substantially across tissue domains. Auricular cartilage has reached first-in-human implantation, although its registered AuriNovo study was terminated after two participants; articular cartilage has limited uncontrolled human evidence; bone and craniofacial applications remain dominated by advanced preclinical work and acellular clinical devices; skin has robust preclinical evidence but lacks randomized comparison with standard grafting; and thick vascularized tissue remains constrained by coupling macrovessels to durable microvascular networks [13,65,71,72,73]. Reviews of the surgical translation pathway emphasize that earlier instances in which regulatory oversight was bypassed, most notably non-validated tracheal implants, produced catastrophic outcomes and now serve as cautionary precedents underscoring the necessity of parallel development of manufacturing, regulatory science, and surgical training [92,93]. Implantable cell-laden bioprinted tissues remain investigational, and no implantable cell-laden bioprinted tissue with FDA marketing approval was identified in the literature reviewed here [13,94]. Across reconstructive domains, recurring translational deficits include the absence of randomized comparative trials, limited long-term in vivo integration data, and manufacturing models that do not yet fit the personalized, on-demand requirements of reconstructive surgery [57,94]. Translational readiness by tissue application, including construct scale, follow-up, functional endpoints, and regulatory status, is summarized in Table 4.

## 13. Regulatory and Ethical Considerations

Bioprinted living constructs occupy a complex and incompletely resolved regulatory space, because products that contain viable human cells and are intended for therapeutic implantation are generally regulated as advanced therapy medicinal products or combination products subject to the most demanding evidentiary pathways [95,96]. In the United States, the 21st Century Cures Act introduced the Regenerative Medicine Advanced Therapy designation to accelerate development of cell and tissue therapies supported by preliminary clinical evidence; however, bioprinted constructs that involve substantial cellular manipulation or non-homologous use will generally fall outside the minimally manipulated tissue framework and be subjected to the investigational new drug and biologics license pathway, as reflected in the regenerative-medicine guidance issued by the Food and Drug Administration [95,97]. Not all bioprinted products meet this threshold, however, and classification as device, biologic, or combination product ultimately depends on the construct’s primary mode of action, a determination made case by case [69].

Beyond classification, the field faces persistent standardization and ethical challenges. Existing biocompatibility and quality-management standards were not written for cell-laden constructs, and there is no validated potency assay or bioprinting-specific good-manufacturing-practice standard, leaving batch-to-batch variability of cell-laden bioinks inadequately controlled [96]. Ethical concerns identified across the literature include informed consent and the potential commodification of cell sources and pronounced equity-of-access problems arising from the high per-construct cost of patient-specific manufacturing, which is currently incompatible with equitable healthcare delivery [96]. Even when biological performance is adequate, the absence of a streamlined approval pathway for personalized, n-of-1 bioprinted implants will continue to constrain clinical adoption.

Printer-to-printer variability, cell-source heterogeneity, and bioink-batch differences further complicate release of living constructs. Identical digital designs printed on different hardware can yield different filament diameters, crosslinking densities, and cell recoveries. Autologous cell isolates vary by donor age, comorbidity, and expansion protocol, and natural bioinks vary by lot. Sterility assurance for open-path extrusion, validated potency and identity assays, and good manufacturing practice (GMP) release criteria remain incompletely specified for n-of-1 reconstructive products. Without interlaboratory reproducibility studies that lock process parameters to functional outcomes, personalized bioprinted implants will be difficult to review as a coherent product class [96].

## 14. Emerging Directions and Future Perspectives

Several emerging technologies are being explored in reconstructive bioprinting. Four-dimensional bioprinting, in which printed constructs undergo programmed shape change after fabrication, offers a route to the complex anatomical contours required for auricular and nasal reconstruction that are difficult to achieve by direct extrusion, exploiting cell-generated or stimulus-responsive forces to guide post-print morphogenesis. Demonstrated morphogenesis to date remains limited to comparatively simple folding and rolling rather than the full complexity of an auricle. Joshi et al. reported 4D-printed multifunctional hydrogels that function as flexible strain sensors and nerve conduits, extending 4D design toward innervated reconstructive targets [46]. Separately, Choudhury et al. described a near-infrared-responsive, deployable and self-fitting 4D-printed bone scaffold that recovered cranial defects in rabbits; that construct is a shape-memory acellular scaffold rather than a cell-laden bioprinted tissue [14]. The integration of artificial intelligence represents a second major direction; within a quality-by-design framework, machine-learning models have been applied to predict bioink printability from rheological properties, optimize print parameters such as nozzle speed, pressure, and temperature, and detect fabrication failures in real time through computer vision, although the poor generalizability of models trained on single-laboratory datasets currently limits their regulatory utility and motivates standardized benchmarking and federated approaches [98]. This is significant for reconstructive surgery specifically, where the n-of-1, patient-specific nature of each construct makes reproducible, predictable manufacturing a prerequisite for both safety and regulatory acceptance. Bioprinting is also converging with organ-on-chip technology, enabling vascularized, multicellular microphysiological models that share vascularization as their central unresolved challenge; in the near term, such patient-specific tissue-on-chip models offer a more tractable translational application than implantable organs [99]. Intraoperative and handheld in situ bioprinting, in which the operative field itself becomes the fabrication environment, represents a further near-term direction that bypasses the logistical burden of ex vivo manufacturing and graft transfer, though sterility, bioink stability at body temperature, and the regulatory classification of intraoperatively manufactured products remain unresolved for human use [58].

**Figure 1:**
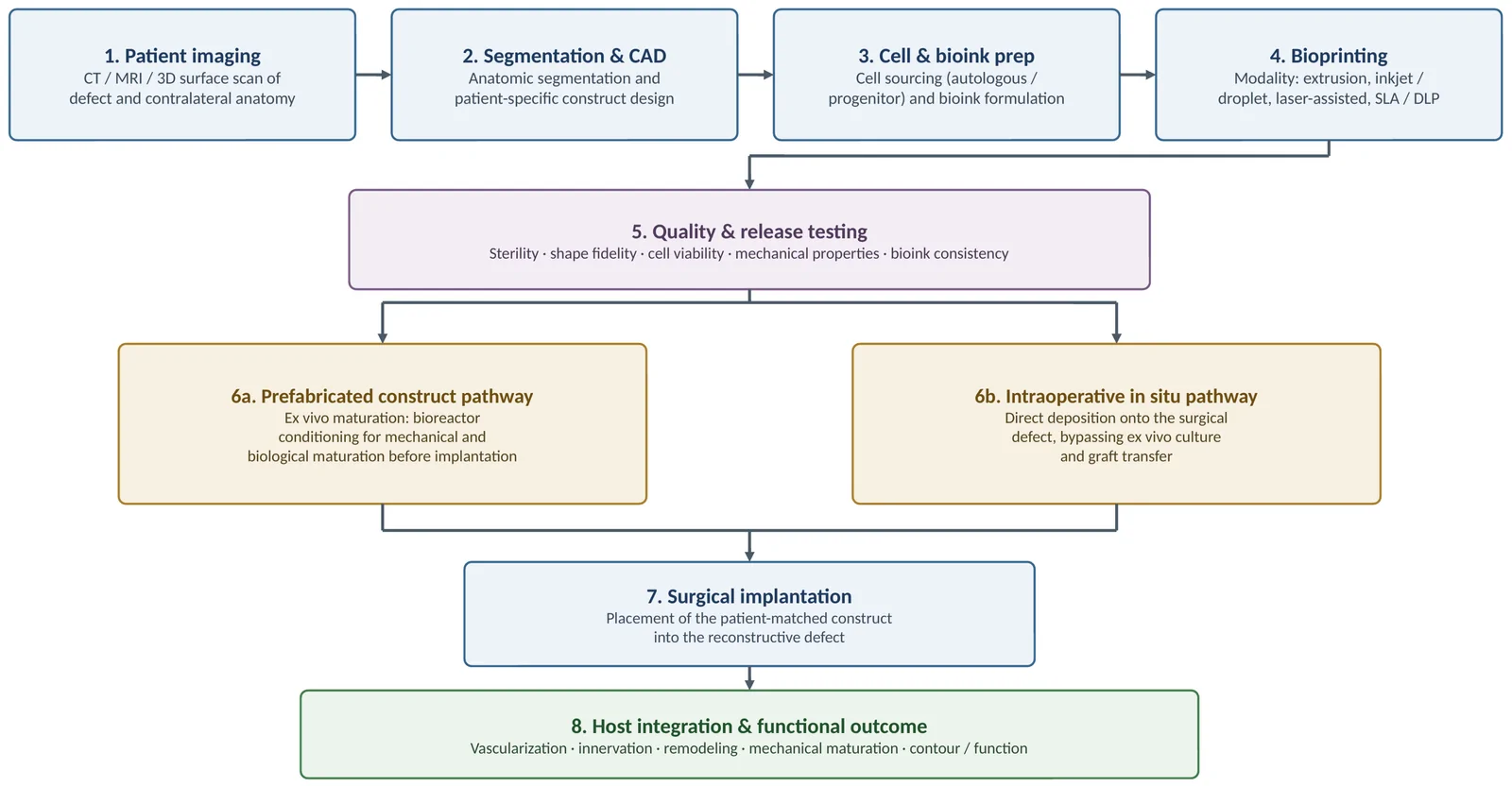
Workflow of 3D bioprinting for reconstructive surgery.

Across these approaches, constructs are becoming increasingly multicellular, anatomically specific, and process-controlled [100]. Yet the principal obstacles remain consistent across tissue domains: vascular integration of thick constructs, mechanical maturation of load-bearing tissues, standardization and good-manufacturing-practice scale-up of cell-laden bioinks, and establishment of a coherent regulatory pathway for personalized living implants. Further translation will require coordinated advances in materials science, vascular biology, surgical practice, and regulatory science. The first investigational human implantations mark an early stage rather than the culmination of clinical translation; rigorous long-term outcome data will be essential to define the clinical role of 3D bioprinting in reconstructive surgery. The overall workflow is illustrated in Figure 1.

Patient-specific imaging and computer-aided design guide construct development, followed by cell sourcing, bioink formulation, and selection of an appropriate bioprinting modality. After quality and release testing, constructs proceed either through an ex vivo prefabricated pathway with bioreactor maturation or through intraoperative in situ bioprinting. Both approaches culminate in definitive reconstruction, followed by host integration, including vascularization, innervation, remodeling, mechanical maturation, and restoration of contour and function. The quality-control checkpoint and dual translational pathways emphasize the manufacturing, regulatory, and clinical considerations discussed throughout this review.

## Figures and Tables

**Table 1:** Representative Recent 3D Bioprinting and Related 3D-Printed Scaffold Studies Relevant to Reconstructive Surgery (2021–2026). Studies were selected to represent distinct fabrication strategies, clinically relevant models, comparative or functional outcomes, and translational benchmarks; acellular comparators are identified explicitly and are not treated as cell-laden bioprinting.

Tissue Domain	Study	Bioprinting Modality	Bioink/Biomaterial	Cell Type(s)	Model System	Key Findings	Ref.
Skin	Jorgensen et al. (2023)	Extrusion-based	Fibrinogen/collagen	Keratinocytes, melanocytes, fibroblasts, HUVECs, follicle dermal papilla cells, pre-adipocytes	Murine and porcine full-thickness wound	Six-cell-type trilayer construct with epidermis, dermis, and hypodermis; rapid vascularization, rete ridge formation, and reduced fibrosis in porcine model	[23]
Skin	Chen et al. (2023)	Robot-assisted in situ	GelMA (photocrosslinked)	Epidermal stem cells, skin-derived precursors	Murine full-thickness wound	Wound healing with regeneration of hair follicles, sebaceous glands, and blood vessels resembling native skin	[24]
Skin	Zhang et al. (2024)	Extrusion-based	GelMA/HAMA/fibrinogen	Human adipose-derived microvascular fragments, human fibroblasts	Murine full-thickness wound	Vascularized bionic dermis; promoted epidermal regeneration, dermal collagen maturation, and accelerated wound healing in vivo	[25]
Skin	Jin et al. (2021)	Extrusion-based (sequential)	Acellular dermal matrix/GelMA	HaCaT keratinocytes, fibroblasts, HUVECs	Murine full-thickness wound	Full-thickness skin model with a vascular network; improved re-epithelialization, ECM secretion, and angiogenesis versus GelMA-only controls	[26]
Cartilage/Bone	Yang et al. (2025)	Digital light processing (DLP)	Methacrylated type I/II collagen, hyaluronic acid, mineralized collagen	Bone marrow-derived mesenchymal stem cells	Rat osteochondral defect	Tri-layer gradient osteochondral organoid with covalent interlayer bonding; near-complete cartilage and subchondral bone restoration at 12 weeks	[27]
Cartilage/Bone	Pitacco et al. (2023)	Extrusion-based (PCL-reinforced)	Fibrin-based bioink with a PCL frame	Human mesenchymal stem cells	Rat femoral defect; subcutaneous nude mouse	PCL-reinforced hypertrophic cartilage template underwent endochondral ossification with vascularization and bone formation comparable to or exceeding BMP-2-loaded collagen scaffolds	[28]
Bone	Machour et al. (2025)	Hybrid extrusion	PLGA/hydroxyapatite/PEG microparticles co-printed with an ECM-based hydrogel	Osteogenic and endothelial cells	Rat femoral defect	Thermosensitive microparticles sinter at 37 °C to form a stiff, porous scaffold; superior bone regeneration versus acellular controls, with vascular network formation	[29]
Bone	Kim et al. (2023)	Extrusion-based	Thermo-responsive poly(organophosphazene) nanocomposite loaded with BMP-2/TGF-β1	Acellular (growth factor-loaded)	Rat calvarial defect	Bioink mechanical properties tunable across 5–37 °C; sustained dual growth-factor release; significantly improved bone regeneration versus controls	[30]
Bone	Jiang et al. (2026)	Extrusion-based	GelMA blended with giant salamander (Andrias davidianus) skin-secretion mucin	Osteoblasts	Rat cranial defect	12.54% new bone volume fraction versus 9.82% for GelMA-only control at 8 weeks; enhanced ALP activity and osteogenic gene expression	[31]
Bone	Loukelis et al. (2025)	Extrusion-based	Gellan gum/polyvinyl alcohol/nano-hydroxyapatite	Pre-osteoblastic cells	In vitro	nHA incorporation improved printing accuracy and reduced biodegradation rate versus gellan gum/PVA control; upregulated osteogenic markers	[32]
Vascularized bone	Fang et al. (2023)	Extrusion-based (granular bioink)	Granular aggregate-prevascularized (GAP) bioink (ECM-based)	Prevascularized mesenchymal spheroids (mesenchymal stem cells, endothelial cells)	In vitro	Cell density of approximately 1.5 × 10^8^ cells/cm^3^ with high shape fidelity; prevascularized spheroids self-organized into an interconnected vascular network via angiogenic sprouting	[33]
Vascularized tissue	Yeo et al. (2026)	Intraoperative bioprinting with surgical micropuncture	Cell-laden bioink	Rat aortic endothelial cells	Rat hindlimb	Micropuncture combined with endothelial-cell-laden bioink produced a 1.8-fold increase in vessel density, 2-fold increase in vessel length, and 2.5-fold increase in PECAM-1 expression at day 40 versus bioink alone	[34]
Vascularized tissue	Muthusamy et al. (2021)	Extrusion-based (multi-head)	Collagen type I/xanthan gum	Endothelial cells, fibroblasts	In vitro	Spatially patterned endothelial cells sandwiched between fibroblast layers self-organized into interconnected capillary-like networks after printing	[35]
Facial reconstruction	Moncal et al. (2021)	Hybrid intraoperative (extrusion + droplet)	Osteogenic hard-tissue ink; separate skin-cell-laden soft-tissue bioink	Osteogenic cells; skin cells	Rat calvarial and full-thickness skin defect	Early demonstration of single-session, composite hard/soft intraoperative bioprinting; substantial bone coverage and wound closure within weeks	[16]
Facial reconstruction	Yan et al. (2023)	3D-printed porous scaffold	Poly(glycerol sebacate) (PGS) elastomer scaffold	Acellular	Rabbit mandibular 8-mm critical-size defect	PGS scaffolds promoted macroscopic bone healing; new-bone width and thickness were greater than empty controls at 6 weeks, with no obvious infection or purulence.	[12]

**Table 2:** Bioink Selection Guide for Reconstructive Bioprinting.

Bioink/Biomaterial	Mechanical/Biological Role	Crosslinking or Processing	Best Reconstructive Applications	Main Advantages	Key Translational Limitations
Collagen type I	Primary structural/bioactive component of dermis; soft, cell-instructive matrix	Thermal self-assembly (pH- and temperature-driven fibrillogenesis)	Dermal layer of skin constructs	Native dermal ECM component; broadly supportive of fibroblast and keratinocyte attachment in reported studies	Mechanically weak and slow-gelling; unsuitable alone for load-bearing constructs; viability figures are formulation- and cell-type-dependent
Fibrin	Provisional wound-matrix analog; carrier for cells and growth factors rather than a long-term structural material	Thrombin-mediated enzymatic polymerization (seconds, at physiological conditions)	In situ skin deposition; growth-factor delivery vehicle in bone applications	Rapid, physiologic gelation compatible with intraoperative and in situ use	Degrades quickly and has limited standalone mechanical integrity; not suited to load-bearing use
Alginate	Shape-templating hydrogel; not inherently cell-adhesive	Ionic crosslinking (Ca^2+^/CaCl_2_)	Cartilage bioinks (typically PCL-reinforced); sacrificial/support materials	Rapid gelation and good shape fidelity; low cost and well characterized	Lacks native cell-adhesion motifs (requires RGD functionalization or gelatin blending) for durable cell attachment
Gelatin methacryloyl (GelMA)	Tunable-stiffness, cell-adhesive hydrogel; among the most extensively characterized bioinks	Photocrosslinking (UV/visible light with a photoinitiator)	Skin, cartilage, and vascular constructs; frequently used as a base component in composite bioinks	Stiffness tunable over a wide range; retains gelatin’s native cell-adhesive sequences	Photoinitiator exposure carries a cytotoxicity risk that increases with concentration and light dose
Hyaluronic acid/methacrylated HA (HAMA)	Contributes anti-inflammatory signaling cues and supports a chondrogenic phenotype	UV crosslinking after methacrylation (unmodified HA is not readily printable)	Cartilage bioinks, typically as a blend component	Bioactive signaling relevant to cartilage biology; improves printability and filament stability when blended	Degrades rapidly and has little mechanical strength in unmodified form; rarely used as a standalone bioink
Decellularized extracellular matrix (dECM)	Retains tissue-specific growth factors and adhesion ligands not reproducible with single-component hydrogels	Thermal gelation following decellularization and enzymatic digestion	Tissue-specific bioinks, e.g., adipose, cartilage (including auricular), and cardiac constructs	Supports tissue-specific differentiation and gene expression signatures in reported comparisons with single-component hydrogels	Lot-to-lot variability from donor tissue and decellularization processing; limited standardization across sources and protocols
Silk fibroin	Broad, tunable mechanical range spanning soft-hydrogel to stiffer regimes	Enzymatic (fast-setting) crosslinking or controlled β-sheet crystallization	Patient-specific structural or auricular/nasal implants	Wide achievable stiffness range; capable of forming durable, memory-shape constructs	Processing is comparatively complex, and mechanical properties are sensitive to crystallinity control
Polycaprolactone (PCL)	Load-bearing structural scaffold; not a cell-delivery bioink in itself	Melt extrusion and thermoplastic solidification (no chemical crosslinking step)	Structural reinforcement of bone, cartilage, and composite hydrogel scaffolds	High mechanical strength with a degradation profile tunable over roughly two to four years	Not intrinsically bioactive; requires co-deposition with a cell-laden hydrogel to support cell delivery

**Table 3:** Comparison of bioprinting modalities used or proposed for reconstructive applications.

Modality	Typical Resolution	Post-Print Viability	Scalability	Speed	Relative Cost	Main Limitation
Extrusion	100–400 µm	High if shear is controlled	Good for cm-scale parts	Moderate	Low–moderate	Limited capillary resolution; hydrogel–stiffness trade-off
Inkjet	30–50 µm	High for low-viscosity inks	Poor for thick tissues	High	Low	Viscosity ceiling; weak mechanical competence
Laser-assisted	10–50 µm	High	Limited	Low–moderate	High	Cost, throughput, and limited construct thickness
Vat/DLP	10–50 µm	Variable; photoinitiator toxicity	Moderate	High for a given volume	Moderate–high	Cytotoxic chemistry; few cell-laden formulations
Embedded/FRESH	20–200 µm	High for soft collagen	Limited by bath removal	Low–moderate	Moderate	Support-bath processing; limited surgical-scale throughput
Aspiration-assisted	Spheroid scale, ~200–400 µm	High for prefabricated spheroids	Poor	Low	Moderate–high	Culture time; poor patient-scale coverage
Chaotic	Internal microscale layers	High in reported hydrogels	Moderate filament throughput	High for internal architecture	Moderate	Hydrogel-bound; no anastomosable vessels
Coaxial/microfluidic	Core–shell, tens–hundreds µm	High when shear is managed	Moderate	Moderate	Moderate	Channels are not surgically anastomosable vessels

**Table 4:** Translational readiness of 3D bioprinting by reconstructive tissue application.

Domain	Skin	Articular Cartilage	Auricular/Nasal Cartilage	Bone/Osteochondral	Craniofacial Composite	Vascular Soft Tissue
Highest evidence	Controlled preclinical	Early human, single arm	First-in-human investigational; trial terminated	Advanced preclinical; isolated cases	Acellular implants plus preclinical composites	Small-animal bioprinted anastomosis; large-animal hybrid grafts
Model	Murine, porcine; robotic in situ swine	Rodent/rabbit; human knee study	AuriNovo Phase 1/2a (n = 2; terminated)	Rodent, rabbit; ITOP-scale mandible or calvarium	Imaging-guided animal models	Rat conduits/flaps; porcine/primate hybrid grafts
Construct scale	Small patches; not major-burn area	Small plugs; patient-specific knee grafts	Patient-specific ear scaffold	Up to patient-scale in animals	Patient-specific acellular; smaller cell-laden	Centimeter scale; 10 cm hybrid graft
Follow-up	Days to weeks	12 months (small human study)	No results posted	Months in selected studies	Variable	60 days (fully bioprinted rat); >4 years (hybrid primate)
Functional endpoint	Re-epithelialization; limited vascular inosculation	Clinical/MRI/histology; no randomized comparator	Contour; no posted trial results or published comparative outcomes	Osteogenesis; limited mechanical testing	Contour and bone fill; sensation not restored	Perfusion and surgical anastomosis in selected models
Large-animal evidence	Porcine wound models	Sparse	Limited	Selected large-defect models	Limited for living composites	Hybrid grafts in pigs and rhesus monkeys
Human evidence	No completed RCT versus STSG	Single-arm study (n = 10)	2 enrolled; trial terminated	Case reports, not trials	Acellular CaP/PEEK/titanium in use; living composites not	None
Regulatory status	Investigational only	Clinical study; no approved product	Orphan/rare-pediatric designations; no marketing approval	No approved cell-laden implant	Acellular devices are not bioprinted tissues	Preclinical
Main remaining barrier	Scale, contamination, head-to-head versus autograft	Controlled comparisons; load durability	Durability and growth versus rib cartilage	Vascularized core; GMP cell expansion	Composite vascularization and sensation	Scale fully bioprinted vessels; integrate capillary beds

## Data Availability

No new data were created or analyzed in this study. Data sharing is not applicable to this article.
